# Investigating the Effects of Ultraendurance Running on Athletes' Heart Rate and Blood Pressure

**DOI:** 10.7759/cureus.58923

**Published:** 2024-04-24

**Authors:** Steven B Hammer, Frederick Strale Jr., Timothy B Williams, Shantele L Kemp Van Ee, James W Agnew

**Affiliations:** 1 Medical and Exercise Physiology, Indian River State College, Fort Pierce, USA; 2 Biostatistics, The Oxford Center, Brighton, USA; 3 Dr. Kiran C. Patel College of Osteopathic Medicine, Nova Southeastern University, Fort Lauderdale, USA; 4 Anatomy and Physiology, Indian River State College, Fort Pierce, USA

**Keywords:** ultrarunning, ultramarathon, blood pressure response, orthostasis, ultraendurance

## Abstract

Background

While the effects of exercise on the cardiovascular system are well documented, ultra-endurance sports involve distances beyond the scope of traditional marathons and have grown in popularity at a staggering pace in recent years. While short-term high-intensity exercise stimulates sympathetic rises in heart rate (HR) and blood pressure (BP), the depletion of fluid and electrolyte reserves characteristic of ultra-endurance sports may contribute to decreases in overall BP after the race. If decompensation of the autonomic safety net occurs, orthostatic hypotension as a result of fluid loss during an event may cause fatigue, dizziness, syncope, or collapse.

Methodology

Subjects were recruited by emails sent to race participants and at pre-race meetings, and no participants were excluded from the study. We observed BP and HR changes in subjects before and after ultramarathon activity in both supine and standing positions over multiple races of variant length and terrain from 50 to 240 km from 2013 to 2018. Participants entered races in Florida, with a mean age of 43.8 and an average body mass index (BMI) of 21.2. In addition to pre-race and post-race measurements, positional post-race BPs and HRs were analyzed for orthostatic trends.

Results

Of those who participated, 140 completed the events and post-race HR and BP measurements were recorded. The mean systolic blood pressure (SBP) increase from pre-race to post-race standing was 21 mmHg, while the mean diastolic blood pressure (DBP) rise was 13 mmHg. While in a supine position, there was a 15 mmHg increase in SBP from pre-race to post-race, along with a 7 mmHg rise in diastolic pressure. Post-race supine to standing average BP change was insignificant. In the supine position, the mean HR increased by 20 beats per minute (bpm) after the race and by 27 bpm while standing. After the race, the average increase in HR supine to standing was 15 bpm.

Conclusions

The SBP changed much more notably than diastolic pressures likely due to the increase in stroke volume associated with the sympathetic response during exercise. HR values also climbed as a result of exercise stress in the setting of catecholamine release, and the combined influence contributed to increased cardiac output despite water and electrolyte loss during the event. Post-race, no trends of orthostatic hypotension were noted either with HR or BP when rising from a supine position. The significance of the contribution of fluid intake during the race to compensatory mechanisms under neural control requires further study.

## Introduction

While the impacts of exercise on the cardiovascular system are well known, ultra-endurance competitions are a relatively modern development with a staggering increase in participation of 345% over the last decade [1]. Blood pressure (BP) regulation is complex and multifactorial, with some debate concerning the contribution of sympathetic outflow versus renal regulation for long-term control [2]. Short-term athletic events are the impetus for sympathetic contribution to hemodynamic stability, while long-term events likely utilize both renin and sympathetic control [3]. Ultra-endurance athletes attempt to account for water losses through increased intake throughout the race, though overconsumption can lead to dangerous hyponatremia [4]. Consumption of water during the event is the runner’s prophylaxis against orthostatic hypotension, an occurrence with dehydration that impedes balance, endurance, and strength and can ultimately cause collapse [5]. Orthostatic hypotension can be objectively measured by serial vital signs, noting a decrease in BP from supine to standing of 20 mmHg or greater and/or a diastolic drop of 10 mmHg or greater [6]. After rising from a supine position, an observed HR increase greater than 30 beats per minute suggests hypovolemia [7]. By measuring pre-race and post-race BP and heart rate (HR) values, the cardiovascular impact of exertion on the runner can be observed. Looking at post-race supine and standing BPs, any trend of orthostatic hypotension can be noted.

## Materials and methods

The research was approved by the Institutional Review Board at Indian River State College in Fort Pierce, Florida. Informed written consent was received from each runner pre-race, before participation in the study. BP data were collected at ultramarathons throughout the State of Florida over five years, including the Wild Sebastian 100 (2013, 2014 - spring and fall), SkyDive Ultra 2016, Fort Clinch 2016, Keys 100 (2016, 2017, 2018), and Saint Sebastian 100 (2017, 2018). Subjects were recruited by emails sent to all race participants and at pre-race meetings the day before each race, and no participants were excluded from the study. The races ranged from 50 to 240 km and occurred at various times of the year. Race terrain varied from paved surfaces for the entire race to sand trials.

BP measurements were made with Omron Series 3 wrist BP monitors. Each cuff was tested against a manual sphygmomanometer before daily use and numbered to reduce intraobserver error on each subject’s BP measurements (the subject was tested with the same cuff on the same wrist, pre-race and post-race). Before each event, participants were placed in a supine position for the initial measurement. Immediately after standing, the BP measurement was repeated to see the initial response to an orthostatic challenge. SBP, DBP, and HR were recorded on individual participant forms. After the race, the same procedure was employed. Each participant was brought to the research area immediately after finishing and measurements were taken within 10 minutes afterward. The research area was within 30 meters of the finish line. 

Pulse pressure (PP) and the mean arterial pressure (MAP) were calculated from collected data using their respective formulas: (PP = SBP - DBP) and (MAP = DBP + [0.3 x PP]). Data were entered and analyzed in IBM SPSS Statistics for Windows, Version 27.0 (IBM Corp., Armonk, NY). One-way analysis of variance (ANOVA) distance x BP measure and race x BP measure showed no significant differences by distance or race.

## Results

While races ranged from 50 to 240 km, the majority were 80 and 160 km races, as shown in Table 1. The characteristics of the sampled runners were an average age of 43.8 and a mean BMI of 21.2 while keeping an average pace of 6.4 km/hour. In the supine position, there was a mean systolic rise of 15 mmHg from pre-race to post-race and a 7 mmHg increase in diastolic measurement. While standing, there was a mean systolic increase of 21 mmHg pre-race to post-race and a mean diastolic rise of 13 mmHg. BP changes are shown in Tables 2-3. The mean increase in HR noted post-race is detailed in Table 4.

**Table 1:** Length of races observed and their percentage of the pre-race sample.

Race length	Percentage of the pre-race sample
50 km	16.6
80 km	47.0
100 km	0.9
120 km	3.7
160 km	30.9
240 km	0.9
Total	100.0

**Table 2:** Blood pressure changes pre-race to post-race in standing posture. SBP, systolic blood pressure; DBP, diastolic blood pressure; PP, pulse pressure; MAP, mean arterial pressure

	Upper limit (mmHg)	Lower limit (mmHg)	*z*-score	*P*-value	Standard error	Mean difference	Hedges’ *g*	Relative weight (kg)
SBP	18.60	23.40	17.16	0.0000	1.22	21	1.64	29.47
DBP	11.11	14.89	13.47	0.0000	0.96	13	1.29	64.76
PP	7.11	10.89	9.33	0.0000	0.96	9	0.89	N/A
MAP	13.11	16.89	15.55	0.0000	0.96	15	1.49	N/A

**Table 3:** Blood pressure changes pre-race to post-race in supine position. SBP, systolic blood pressure; DBP, diastolic blood pressure; PP, pulse pressure; MAP, mean arterial pressure

	Upper limit (mmHg)	Lower limit (mmHg)	*z*-score	*P*-value	Standard error	Mean difference	Hedges’ *g*	Relative weight (kg)
SBP	12.53	17.47	11.88	0.0000	1.26	15	1.14	4.61
DBP	5.21	8.79	7.66	0.0000	0.91	7	0.73	18.67
PP	7.21	10.79	9.85	0.0000	0.91	9	0.94	n/a
MAP	8.11	11.89	10.36	0.0000	0.96	10	0.99	n/a

**Table 4:** Mean HR changes during pre-race and post-race serial vital signs. Mean HR increases pre-race to post-race in supine and standing positions. HR, heart rate

Pre-race mean HR change from supine to standing	Post-race mean HR change from supine to standing	Mean HR change pre-race to post-race supine	Mean HR change pre-race to post-race standing
+3 bpm	-3 bpm	+20 bpm	+27 bpm

Regarding serial vital signs taken before and after the race, pre-race HRs climbed an average of 8 bpm, while post-race HRs showed a mean increase of 15 bpm. BPs with postural changes increased by 2 mmHg pre-race when rising to a standing position from supine. Conversely, after the race, the mean BP decreased by 3 mmHg.

## Discussion

BP during ultramarathon events is affected by the vasodynamic stress response of the body under autonomic control [8]. The complicated interplay of catecholamine release, insensible fluid loss, and electrolyte depletion affect a runner’s capability to endure extended-distance events. While these responses vary among individuals, the potential hazards remain the same: depletion of resources may result in hemodynamic instability and collapse. Runners typically employ hypovolemia preventive measures through fluid intake during the event, such as hydration and electrolyte replacement [9].

The increase in HR from pre-race to post-race followed the expected result after exercise-induced catecholamine release [6,10]. The evaluation of postural hypotension post-race was to discern any trend signaling potentially dangerous fluid loss. The mean BP rise as a result of increased stroke volume during the race also followed an expected trajectory, while the post-race supine to standing value showed negligible change with no trend toward orthostatic hypotension.

The average increase in overall BP post-race was mild and compensatory, without obvious danger to healthy vasculature. The increased stress on a normal cardiovascular system appears stable for ultra-endurance events, though age should be considered. It is worth noting the average runner in this study was middle-aged. Pre-existing congenital defects, undetected anomalies, advanced age, and comorbidities must also be carefully regarded when athletes begin training for events requiring sustained stress on cardiovascular resources. Replenishing fluids during such events appears to result in the prevention of orthostatic hypotension due to fluid loss and potential decompensation of autonomic safeguards. 

Limitations

The individual autonomic response is variable and difficult to account for. While every effort was made to gather multiple races and runners over time, the sampled athletes were not equally divided between male and female genders. Information concerning comorbidities or medication use, which may affect the body’s hemodynamic response was not gathered. The longest event measured (240 km) made up a very small proportion of runners evaluated and most of the data gathered came from 80 and 160 km events. This may limit the applicability of cardiovascular response observed to extremely long-running events greater than 200 km. The locations studied were confined to the state of Florida, known for its heat, humidity, and lack of altitude, impacting the body's response to physical stress. The data were not further delineated by the time of year to consider weather patterns and their effects on the physiological response to exercise.

This study does not consider the rise in multi-sport ultra-endurance events, such as Iron Man, and the body’s potentially different response to varied activities over an extended time frame. Orthostatic trends were not observed, but supine to standing measurement occurred immediately and the runners were not observed for the full three minutes after changing positions typically required during serial vital signs [3]. The sample decreased from pre-race to post-race as a function of several athletes not finishing the races.

Future research

Fluid loss should be replaced judiciously to avoid dangerous hyponatremia, and the correct calculation of such based on the individual runner and event should be studied further. Comparing hemodynamics in multi-sport ultra-endurance activities would yield observations concerning the body’s response when different muscle groups are recruited. The vasodynamic response and compensatory mechanisms between differing types of activity and the overall event in comparison to strictly mega long-distance running events should be evaluated.

## Conclusions

There has been a marked increase in participation in ultraendurance events in the past 20 years, with an increasing number of middle-aged athletes. The increased interest among older and potentially vulnerable participants in ultraendurance events with sustained exercise stress warrants careful consideration of cardiovascular risk. The human body safety mechanisms for cardiovascular endurance, including neural and renal responses, appear intact during and after ultraendurance races, as no decompensation was noted in participants. HRs and BPs did not trend to dangerous levels and decompensation due to hypovolemia did not occur. Increases in HRs and BPs during extended periods of exercise appeared to be transient and minor. As with any long-distance exercise, endurance training before events and thorough medical examination to evaluate individual tolerance of races should occur. Through complex mechanisms of autonomic control, the human body is capable of tolerating extended exercise-induced stress with a minimal and short-term impact on a healthy cardiovascular system.
